# *Didymium arenosum*, a myxomycete new to science from the confluence of deserts in northwestern China

**DOI:** 10.7717/peerj.16725

**Published:** 2024-01-08

**Authors:** Shuwei Wei, Shu Li, Pu Liu, Bao Qi, Qi Wang, Yu Li

**Affiliations:** 1 Engineering Research Center of Chinese Ministry of Education for Edible and Medicinal Fungi, Jilin Agricultural University, Changchun, Jilin, China; 2 College of Plant Protection, Jilin Agricultural University, Changchun, Jilin, China; 3 Northeast Normal University, Changchun, Jilin, China

**Keywords:** *Didymium*, Morphology, Phylogeny, Scanning Electron Microscope, SSU rDNA, The life cycle

## Abstract

A new myxomycete species, *Didymium arenosum*, was described based on morphological evidence and phylogenetic analyses. The species was discovered in the arid region at the confluence of the Badain Jaran desert and Tengger desert on the leaves of *Betula platyphylla* and was cultivated in a moist chamber culture. Morphologically, the species is distinguished by the greenish-yellow calcium carbonate crystals on the surface and the spores covered with small warts, some of which are connected into a short line. A phylogenetic analysis of *D. arenosum* strongly supports its classification as a separate clade. The spore to spore agar culture of *D*. *arenosum* requires 23 days, and this study provides a detailed description of its life cycle.

## Introduction

Myxomycetes are a protist group belonging to the Eumycetozoa (Kang et al., 2017; Hehmeyer, 2019). They have played a crucial ecological role in terrestrial habitats and some of those metabolites have also been demonstrated to exhibit antitumor activity, antibacterial activity, and antioxidant activity (Keller & Everhart, 2010; Li et al., 2022; Wang, Li & Liu, 2022). Populations of these organisms are primarily regulated by food-related feeding practices, contributing to the maintenance of species diversity within their biosphere (Foissner & Hawksworth, 2009; Stephenson, Laursen & Seppelt, 2007). The investigation of slime molds in arid regions is mainly limited to certain areas in America, Asia, and Europe due to the difficulty of collections (Estrada-Torres et al., 2009; Lado et al., 2013; Lado et al., 2016; Novozhilov & Schnittler, 2008; Stephenson et al., 2020; Wrigley de Basanta, Lado & Estrada-Torres, 2010; Wrigley de Basanta, Lado & Estrada-Torres, 2012). Nevertheless, these studies have laid the foundation for exploring the diversity of myxomycetes in arid regions (Estrada-Torres et al., 2009; Wrigley de Basanta et al., 2009).

*Didymium* (Didymiaceae) was initially described in the 18th century, and to date, more than 90 species have been reported (Janik et al., 2021; Kirk et al., 2008; Lado, 2023). Previous studies indicated that *Didymium* is more likely to survive in arid areas than in other areas. For instance, nearly 20% of the species of *Didymium* were found in the arid and semi-arid regions of the Canary Islands. In addition, more than 20% of the species were found in the Twakan-Quikateland Valley, a biosphere reserve in Mexico (Estrada-Torres et al., 2009). However, recent studies have shown that most *Didymium* species are found in temperate and tropical climate regions (Wrigley de Basanta et al., 2015; Wrigley de Basanta, Lado & Estrada-Torres, 2008). China has a vast territory and abundant resources, but until now, only Schnittler has investigated the diversity of slime molds in the Tarim Basin of Xinjiang. In the future, it is necessary to explore further the species diversity of slime molds in the arid region of China, including the Gurbantunggut Desert, the Qaidam Basin Desert, and the Kubuqi Desert, etc (Schnittler et al., 2013; Zhang et al., 2020).

Minqin County is situated on the confluence of Badain Jaran desert and Tengger desert, which is an arid area in northwestern China, at 38°03′–39°28′N and 101°49′–104°12′E (Sun et al., 2005). The region undergoes an arid continental climate (Ma et al., 2007) with an annual average precipitation of less than 150 mm and annual evaporation exceeding 2,500 mm (Sun et al., 2007). Evaporation occurs at a rate approximately 20 times greater than precipitation. The elevation ranges from 1,280 to 1,477 m, and the average annual temperature is 8.3 °C (Xue et al., 2015). Vegetation coverage in arid regions is less than 10%, dominated primarily by *Artemisia arenaria* DC., *Ephedra prezewalskii* Stapf, *Haloxylon ammodendron* (C.A. Mey.) Bunge, and *Nitraria tangutorum* Bobr. (Chang et al., 2006). These plants are important for the protection and management of ecosystems in semiarid and arid regions (Wu et al., 2021).

Myxomycetes are rare in arid regions (Schnittler et al., 2013). However, recent research showed that 39 species of slime molds were discovered in arid areas of northwestern China from 2016 to 2018, of which only four belonged to the *Didymium* (Wei et al., 2019). The collection of these myxomycetes were obtained from live tree bark, deciduous, and dead branches, then through moist chamber culture using Cavender’s methods (Cavender, 1995). These findings provide valuable insights into the distribution and diversity of myxomycetes in arid regions, and our research will contribute to the understanding of these fascinating organisms.

Slime molds’ life cycles undergo transformations, progressing from trophic, diploid multinuclear plasmodia to reproductive, haploid fruiting bodies (Keller, Everhart & Kilgore, 2022). It has the capability to directly develop into haploid plasmodium, or it can generate diploid zygotes through heterothallism or homothallism. These then gradually evolve into amorphous, multinuclear plasmodia through continuous mitosis or fusion with other myxamoebae (Keller, Everhart & Kilgore, 2022). However, the detailed morphogenesis of only a few species has been described so far primarily because plasmodium retrieval from some species is difficult, especially regarding the techniques used in culturing plasmodia (Chen et al., 2013; Keller & Schoknecht, 1989a; Keller & Schoknecht, 1989b; Liu, Wang & Li, 2010).

## Materials & Methods

### Morphological studies

**Figure 1:**
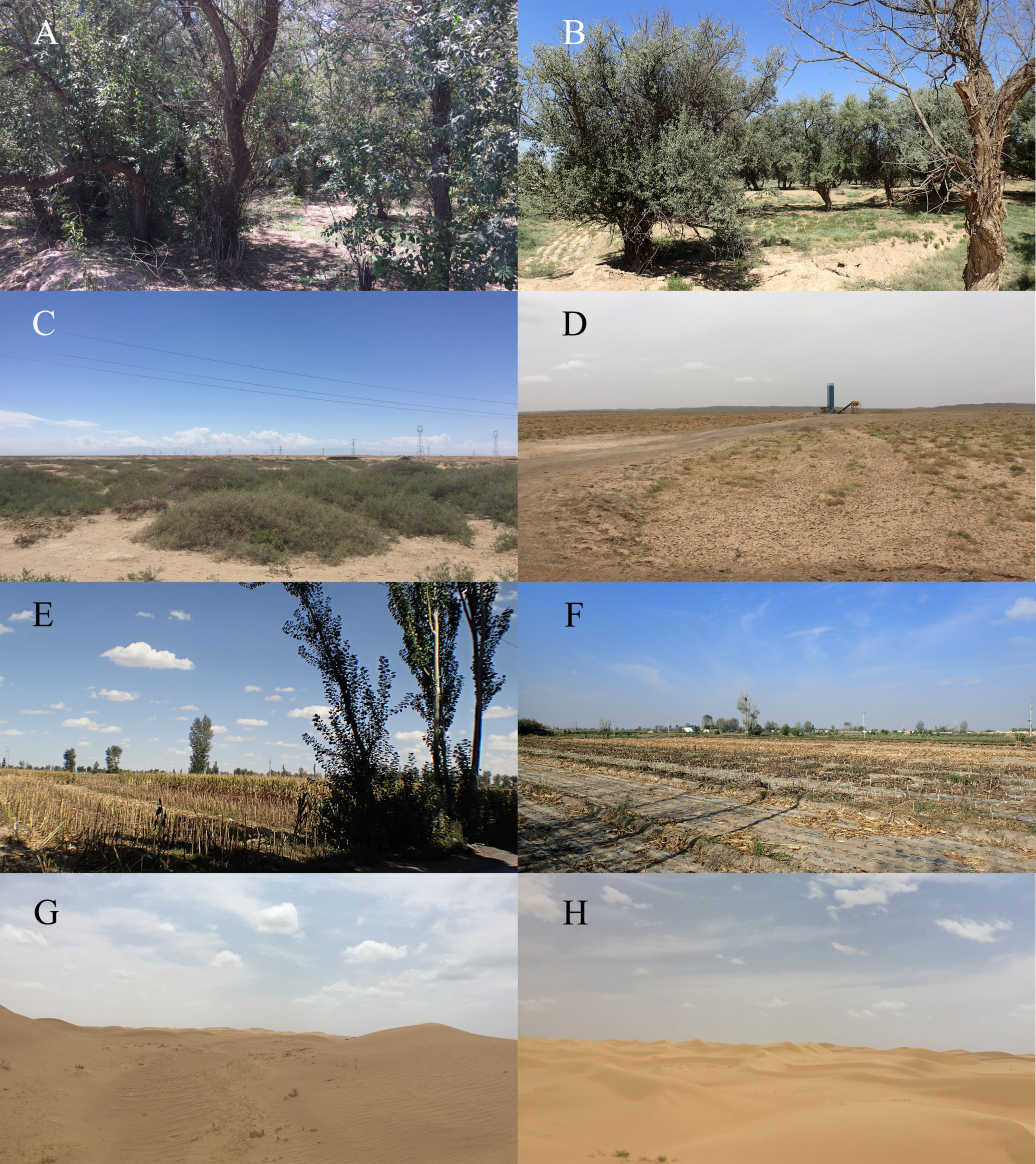
Representative habitats of Minqin County, Gansu province, China. (A, B) The artificial forest; (C, D) the desert transition zone; (E, F) the farmland; (G, H) the desert.

The specimens of *D*. *arenosum* were obtained from leaves collected in Minqin County by moist chamber culture from 2016 to 2018 (Fig. 1). These specimens (HMJAUM15005–HMJAUM15007) were deposited in the Herbarium Mycology of Jilin Agricultural University (HMJAU). Hoyer’s Mounting Medium was used for mounting myxomycete spores. One hundred myxomycete spores were observed and measured using the ZEISS Axioscope 5 and Axiocam 506 color photographic system. The ornamentation was observed and measured using a Hitachi SU8010 Scanning Electron Microscope (SEM) running at 5 kV to examine the ultrastructure (García-Cunchillos, Estébanez & Lado, 2021; He et al., 2022).

### Water agar culture

Water agar cultures were prepared according to the methods previously described by Haskins & Wrigley de Basanta (2008) and Tran et al. (2012), summarized as follows: 20 g agar (Shanghai Sangon, China) was added to 1,000 mL sterilized water, and sterilized for 20 min at 121 °C. After sterilization, the bottle was transferred to the super clean bench, and the water agar medium was poured into 9 cm glass Petri dishes. Subsequently, spore suspensions were inoculated onto the media. Continuous observation the formation of plasmodia.

### Spore germination and single-spore cultivation

Four sporocarps of *D*. *arenosum* were placed in 1.5 mL plastic tubes and 500 µL of sterile water was added to each tube. The tubes were incubated in the dark at 25 °C for 2–12 h until spores germinated. Subsequently, they were added to 2.0% water agar medium with pH of 7.0. Spores were released over the water agar medium. After 48 h, transparent phaneroplasmodia appeared on the culture medium. When the plasmodia began to grow on the cultural medium, these plates were immediately placed in a dark incubator at 22–25 °C. After seven days, we transferred the Petri dish to natural light for incubation to stimulate the formation of sporocarps.

### DNA extraction and PCR amplification

The DNA was extracted from three to five sporocarps, including the type specimen (HMJAUM15007!), using the DNeasy Plant Minikit (QIAGEN, Shanghai, China), following the manufacturer’s instructions. The small subunit ribosomal DNA (SSU rDNA) sequence fragment was amplified *via* PCR amplification, with each reaction mixture containing 2.5 µL 10 × PCR Buffer (Mg2+), 2.5 µL dNTP (2.5 mM/mL), 1 µL each primer, 0.25 µL rTaq (5 U/µL), 0.5 µL DNA template (10 ng/µL) and double-distilled water to a final volume of 25 mL. The primers were Phf1b-A (AAAACTCACCAGGTCCAGAT) and JKr-2 (AGGGCAGGGACGCATTC) (Wrigley de Basanta et al., 2015). The reactions were performed with the following program: initial denaturation at 94 °C for 3 min, 30 cycles of 94 °C for 30 s, 58 °C for 30 s, 72 °C for 2.5 min, followed by 72 °C for 10 min. PCR products were sent to Sangon Biotech Co., Ltd. (Shanghai, China) for sequencing to be directly sequenced using the ABI 3730xl DNA analyzer.

### Phylogenetic analyses

The newly generated sequences obtained in this study have been deposited in GenBank (Table 1). For the datasets, the alignment was generated using the “L–INS–i” strategy of MAFFT v.7.017 (Katoh & Standley, 2013). Before performing phylogenetic analyses, start and end ambiguous sites were removed, and gaps were manually adjusted to optimize the alignment by BioEdit v7.1.3 (Hall, 1999). Maximum parsimony (MP) methods were employed using PAUP 4.0 to generate the trees (Wilgenbusch & Swofford, 2003). Bootstrap analysis was performed with 1000 replications to evaluate the topological confidence of MP trees. Bayesian inference (BI) phylogeny analyses were conducted using Markov chain Monte Carlo (MCMC) with Mrbayes 3.2.6 (Ronquist & Huelsenbeck, 2003).

**Table 1:** Taxa information and GenBank accession numbers of the sequences used in this study.

Taxon	GenBank accession	DNA region	Voucher ID	Length (bp)
*Didymium bahiense*	AB259387	18S	TNS-M-Y-4944	428
*Didymium clavus*	AB259389	18S	JM-4507	423
*Didymium clavus*	AB259390	18S	AK-04172	422
*Didymium clavus*	AB259391	18S	AK-04296	423
*Didymium clavus*	AB259392	18S	TNS-M-Y-17152	422
*Didymium crustaceum*	AB259395	18S	TNS-M-Y-17612	425
*Didymium crustaceum*	AB259396	18S	YY-26183	425
*Didymium crustaceum*	MW404616^*^	18S	20180821050	429
*Didymium crustaceum*	MW404618^*^	18S	20181007025	429
*Didymium crustaceum*	MW404630^*^	18S	20160923065	431
*Didymium crustaceum*	MW404631^*^	18S	20160704011	428
*Didymium dubium*	AB259399	18S	TNS-M-Y-17046	427
*Didymium dubium*	AM231294	18S	K7	1932
*Didymium dubium*	AM231295	18S	K15	1911
***Didymium arenosum***	**MW413352** ^*^	**18S**	**HMJAUM15005**	**419**
***Didymium arenosum***	**MN720571** ^*^	**18S**	**HMJAUM15006**	**432**
*Didymium floccoides*	AB259402	18S	AK-04032	428
*Didymium floccoides*	AB259403	18S	AK-04046	429
*Didymium floccosum*	AB259405	18S	TNS-M-Y-16882	434
*Didymium floccosum*	AB259406	18S	JM-3011	434
*Didymium iridis*	AB259407	18S	JM-S-08	430
*Didymium iridis*	AB259408	18S	JM-643	434
*Didymium laccatipes*	AB259410	18S	AK-F028	428
*Didymium marineri*	AB259413	18S	TNS-M-Y-15365	426
*Didymium megalosporum*	AB259414	18S	JM-S-06	426
*Didymium megalosporum*	AB259415	18S	JM-4509	426
*Didymium nigripes*	AB435333	18S	AK-05137	433
*Didymium nigripes*	AB435334	18S	AK-06080	425
*Didymium nigripes*	AB435335	18S	AK-06100	433
*Didymium nigripes*	AB435336	18S	AK-06110	433
*Didymium nigripes*	MW404632^*^	18S	HMJAUM15003	438
*Didymium panniforme*	AB259428	18S	TNS-M-Y-16880	525
*Didymium squamulosum*	AB435337	18S	AK-06063	433
*Didymium squamulosum*	AB435338	18S	AK-06085	435
*Didymium squamulosum*	AB435339	18S	AK-06119	435
*Physarum roseum*	HE614605	18S	C1	1820

**Notes.**

Newly generated sequences in this study are in bold.

* Sequence provided in the study.

## Results

### Taxonomy

**Table:** 

**Figure 2:**
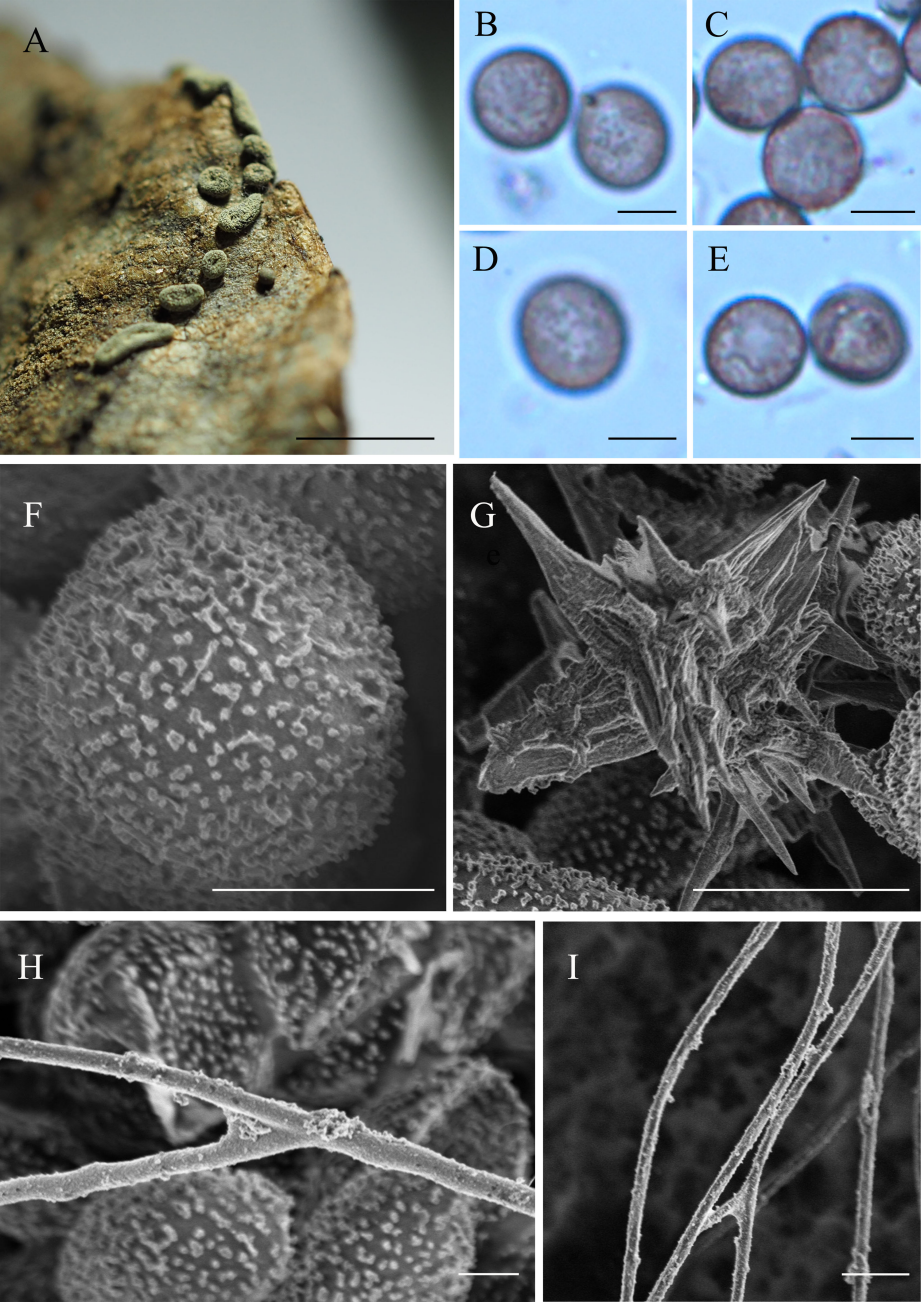
*Didymium arenosum.* (HMJAUM15005). (A) Sporocarps; (B–E) spores by LM; (F) spore by SEM; (G) stellate calcareous crystals by SEM; (H, I) capillitium by SEM. Scale bars: (A) = 2.5 mm; (B–E) = 5 µm; (F) = 5 µm; (G) = 10 µm; (H) = 5 µm; (I) = 10 µm.

***Didymium arenosum* S.W. Wei, Q. Wang & Y. Li, sp. nov.** (Figs. 2A–2I)

MycoBank: MB849855

Etymology: “arenosum” refers to sandy or full of sand, it is derived from the Latin “arena”, which means sandy place.

Holotype: CHINA, Gansu Province, Minqin County, Harvest Township 103°35′E38°53′N, alt. 1317 m, leaves obtained in the moist chamber culture, pH 7.3, 17 Sept. 2016, Shu-Wei Wei, (Holotype HMJAUM15007!, 18S = OR500607).

Sporophores sporocarpic, occasionally fused to form short plasmodiocarps, 0.25–0.5 mm high, dispersed or grouped, spherical or flattened, or umbilicate above, greenish-yellow, pale yellow, or yellowish-brown, composed of stellate calcareous crystals, blackish when the crystals are sparse or absent. Sporocarps are sessile or have a short stalk, scattered, globose to subglobose, pulvinate, and plasmodiocarp elliptical or often curved. The centerline is slightly sunken and grows on a colorless and invisible substrate; sessile sporocarps 0.5–0.9 mm diam, whereas plasmodiocarps with a width of 0.3–2.3 mm, and a thickness of 0.3–0.5 mm. Columella flat, dark yellow, or brown. Hypothallus insconspicuous, membranaceous, and individualized to each sporophore. Capillitium filiform, branched, tenuous, and with few cross connections, colorless to light brown, threads 0.5–2.0 µm diam, enlargements 2.7–3.9 µm diam, smooth but with a granular surface by SEM. Spores free, black in mass, dark brown to brown by LM, globose to subglobose, (7.4)7.8 × 9.8(10.8)–(7.7) 8.3 × 10.6(11.2) µm, densely warted by LM, with evenly distributed bacula by SEM, and the spore ornamentation are small warts, although some rounded to irregular, or forms short lines.

Habitats: leaves of *Betula platyphylla*.

Additional specimens were examined. CHINA, Gansu Province: Minqin County, Harvest Township, 103°35′E, 38°53′N, alt. 1317 m, 13 Sept 2016, Shu-Wei Wei (HMJAUM15005); Suwu Township, 103°6′E, 38°7′N, alt. 1335 m, 17 Sept. 2016, Shu-Wei Wei (HMJAUM15006).

### The life cycle

**Figure 3:**
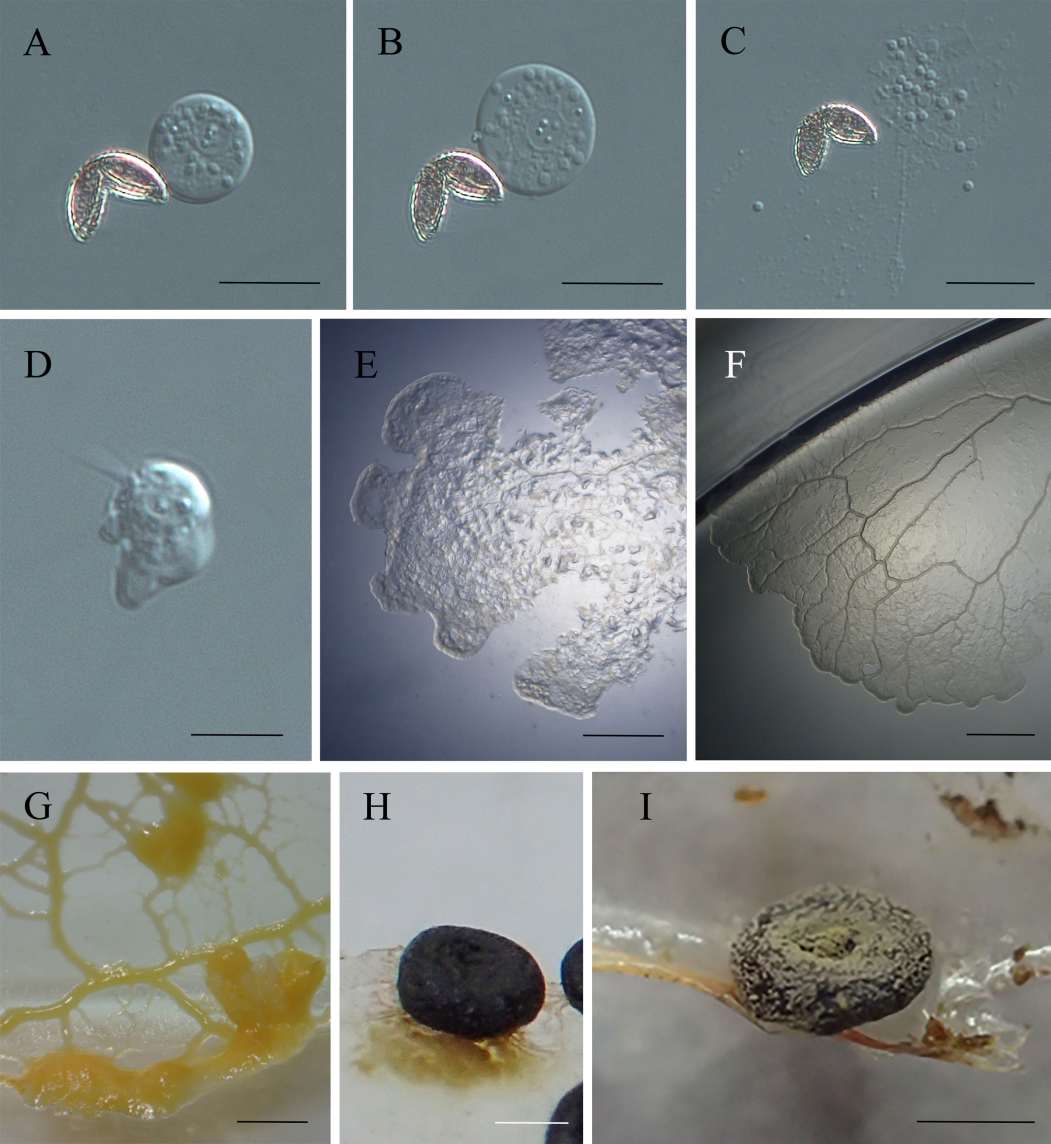
The life cycle of *Didymium arenosum*. (A–C) Spores with V-shaped split; (D) swarm cell; (E) young plasmodia; (F) young plasmodia radiate forward; (G) mature plasmodia; (H) young sporangium; (I) mature sporangium. Scale bars: (A, B) = 10 µm; (C) = 8 µm; (D) = 10 µm; (E) = 0.1 cm; (F) = 0.3 cm; (G) = 0.5 cm; (H, I) = 0.5 mm.

Spore germination occurred *via* the split method creating a V-shaped opening in sterile water (Figs. 3A–3C). After 3 h of suspension culture, the myxamoeba and swarm cells (Fig. 3D) were released from the spore. The swarm cells, which have two flagella at the anterior end, moved rapidly, while shorter projections were often attached to the side and not easily observed. Under strong light, the spores released their internal contents, gradually growing in size (Fig. 3B) before disappearing (Fig. 3C).

Initial plasmodia were observed around 7–15 days after the spore suspension of the swarm cells was transferred to the water agar medium (Fig. 3E). Meanwhile, after 2–4 days of cultivation, the initial plasmodia radiated forward, were colorless, lacked an obvious netted form, and had few branches (Fig. 3F). The thickened reticular mature plasmodium mass at the leading edge could be observed after shading and additional oat feeding after 3–6 days (Fig. 3G). The plasmodia matured, transitioning from milky yellow to brownish-yellow, with a sizeable front sector area and many branches, and distributed on the water agar surface as a network.

The mature plasmodia were cultured at 20–22 °C and exposed to natural light. Within 24–48 h, the mesh-shaped plasmodium began to form sporocarps. The formation of sporocarps varied in time, with some developed within three days, while most remained in the vegetative growth stage or formed scleotia during this period. Incomplete secondary germination without light was possible. With exposure to natural light, the plasmodia masses gradually coalesced, forming more robust veins. The sporangium changed color from milky white to light yellow before gradually blackening (Fig. 3H). When the environment was appropriately dry, yellowish-brown or yellow-green calcareous crystals formed on the surface of the sporocarps (Fig. 3I).

### Phylogenetic analyses

**Figure 4:**
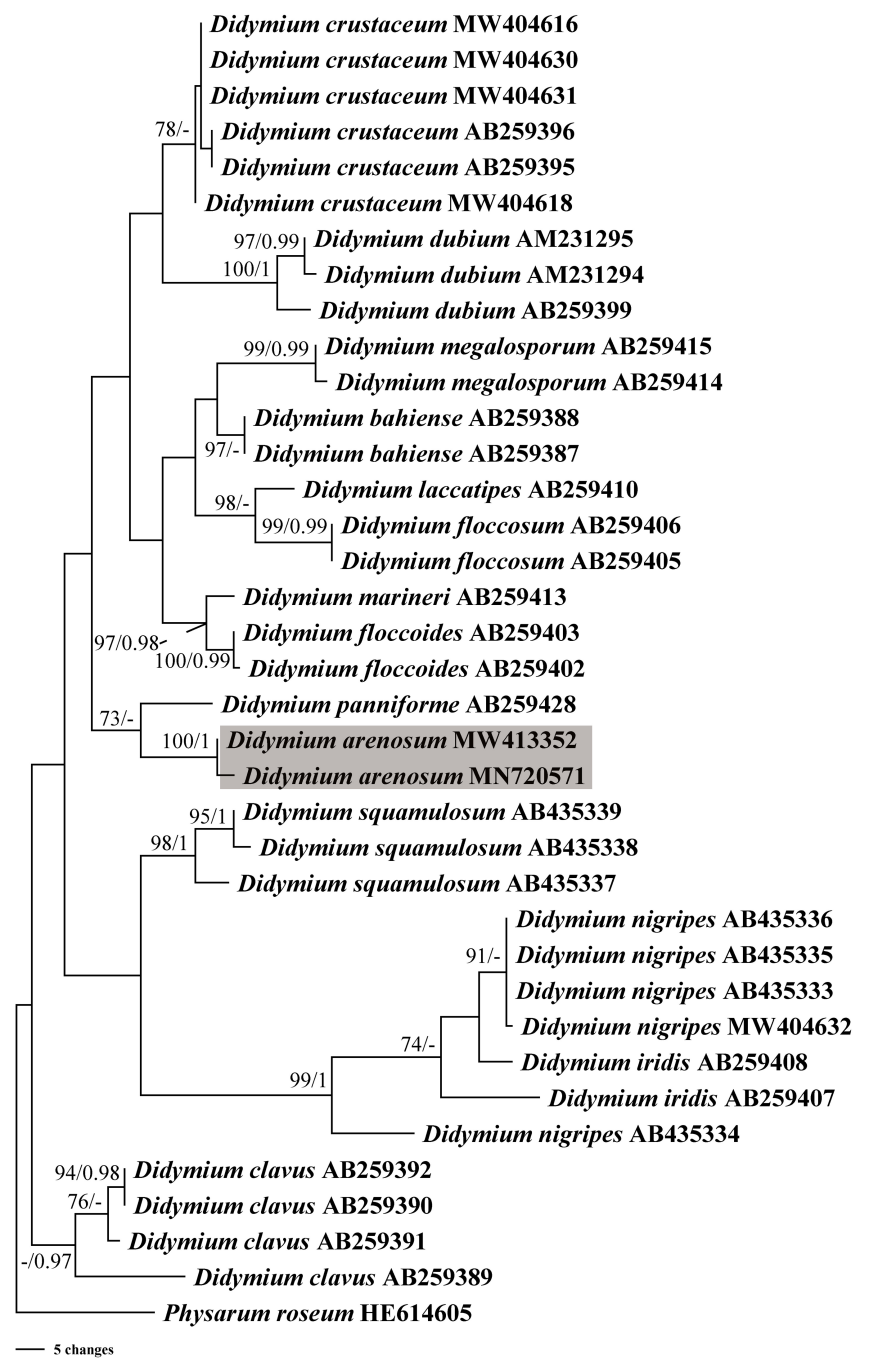
Bayesian inference (BI) and maximum parsimony (MP) phylogenetic analysis based on the SSU rDNA sequences of *Didymium* species. Branches are labeled with Bayesian posterior probabilities greater than 0.95 and maximum parsimony bootstrap support more significant than 70%. The newly generated sequences are bolded, and the new species are indicated in a shadow of gray.

In this study, we obtained the SSU rDNA sequences of *D*. *arenosum*, *D*. *crustaceum* Fr., and *D*. *nigripes* (Link) Fr. A total of 36 sequences with 403 positions were analyzed phylogenetically, with a myxomycete from *Physarum roseum* Berk. & Broome as an outgroup. The GTR+I+G nucleotide substitution model was found to be the best fit. The MP and BI trees had similar topologies, with only minor differences in a few nodes with low MP/BI support. Our phylogenetic analyses revealed that the new species *D*. *arenosum* forms an independent branch cluster closely related to *D. panniforme* J. Matsumoto (Fig. 4).

## Discussion

Based on the morphological and phylogenetic analyses, this study discovered a new species in the arid region, and completed its life cycle on water agar culture. Morphologically, the sporangium of *D*. *arenosum* was smaller than *D*. *ochroideum* G. Lister (Lister, 1931), whereas *D*. *arenosum* was greenish-yellow, pale yellow, or yellowish-brown, while *D. ochroideum* was light orange-brown. In macro-morphology, *D. arenosum* was similar to *D*. *obducens* P. Karst, but the color of peridium was different, and *D. obducens* was pale brownish ochraceous. In micro-morphology, the capillitium of *D. obducens* was light brown, and the tips were usually darker. Furthermore, *D. obducens* can be distinguished by the larger spores (12–14 µm). Unlike *D*. *arenosum,* the surface of *D*. *panniforme* (Mastsumoto & Deguchi, 1999) was covered with orange calcium carbonate crystals, similar to the new species. There were many ridges on the surface of spores, most of that were connected by an incomplete network. In contrast, *Didymium arenosum* has irregular spines and warts which can be combined into short lines. The sporangium of *D. arenosum* and *D*. *tussilaginis* (Berk. & Broome) Massee are morphologically similar, occasionally fused to form plasmodiocarps, which are difficult to distinguish with the naked eye. However, the inconspicuous columella of *D*. *tussilaginis* and the larger spores (12–13 µm) help distinguish it from the new species (Baba et al., 2021). Within the myxomycetes, phylogenetic research is still at a relatively early stage, and our phylogenetic analyses suggested that *D*. *arenosum* has close affinities with *D. panniforme* (Fig. 4), consistent with the morphological study (Leontyev & Schnittler, 2022).

Previous studies have shown that the myxomycetes cultivated in the moist chamber could be found in three months (Wrigley de Basanta et al., 2017). However, it only needs half a month to form the sporangium in this study. The likely explanation is that the species mainly grows in the arid regions. Therefore, the microhabitat changed suddenly in the presence of favorable growing conditions in the moist chamber, resulting in the growth rate of the species significantly accelerated.

In the moist chamber cultures of *D*. *arenosum*, the substrate pH ranged from 6.4 to 7.6, with an average pH of 7.0. This range is similar to the circumneutral pH of the agar medium on which it completed its life cycle from spore to spore. Several studies have demonstrated that substrate pH affects the growth of moist chamber cultures (Wrigley de Basanta, 2000; Wrigley de Basanta, 2004). However, some species have adapted to basic media in arid regions. For example, *Didymium wildpretii* Mosquera, Estrada, Beltrán-Tej., D. Wrigley & Lado grows on substrata with pH values of 7.5–10, and *Licea succulenticola* Mosquera, Lado, Estrada & Beltrán-Tej also grows on a substrate with a higher pH (Lado et al., 2007; Mosquera et al., 2002). The differences in pH selection might reflect the different organisms during a succession of substrate decay rather than direct effects on the myxomycetes. Both bacteria and yeasts have been shown to have high levels of specialization in the host plant (Wrigley de Basanta, Lado & Estrada-Torres, 2008).

During the life cycle of *D*. *arenosum*, germination occurred through a V-shaped split within 3 to 72 h, releasing swarm cells with one long and one short flagellum that swam in the sterilized water. This germination mode is common among many species of *Didymium* (Gao et al., 2017; Ishibashi et al., 2001; Lado et al., 2007). Similarly, the life cycle of *D*. *arenosum* and *D. xanthopus* (Ditmar) Fr. required light treatment (Gray, 1938). Without light, the plasmodia of *D*. *arenosum* would gradually dissolve or form sclerotia in the medium. The life cycle of this species proceeds in a closed system with constant humidity and temperature, so the sclerotia formation was not a response to drying, it might be due to self-protection mechanism. Therefore, the conditions for sporangium formation in *D*. *arenosum* depend on external conditions and the abundance of nutrients. Overall, our findings suggest that the life cycle of *D*. *arenosum* is similar to that of other *Didymium* species, and the formation of sporangium requires light. Additionally, the formation of sclerotia in agar culture may be influenced by nutrient availability rather than dampness degree. Further research is needed to elucidate the mechanisms underlying these processes in *D*. *arenosum* and other related species.

The germination of *D*. *arenosum*, within 72 h, which is similar to another species *D. infundibuliforme* Wrigley de Basanta et al. (2009). *Didymium squamulosum* has a life cycle of only 12 days (Zhu et al., 2019), likely influenced by its extensive distribution and need to occupy sufficient ecological niches in the natural ecosystem. *Didymium infundibuliforme* completed its life cycle in about 50 days (Wrigley de Basanta et al., 2009), while *D. umbilicatum* D. Wrigley, Lado & Estrada from arid regions of Mexico, takes at least 51 days when cultured (Wrigley de Basanta et al., 2009), and *D. wildpretii* takes about 28–56 days (Lado et al., 2007). Blackwell & Gilbertson (1980) suggested that a shorter life cycle would be more adaptive in extreme desert conditions. However, the ability and propensity of these species to rapidly generate sclerotia could serve as a more reliable survival mechanism against sudden environmental changes, and these dormant periods would prolong its likelihood of survival.

## Conclusions

Through morphological studies, life cycle, and phylogenetic analyses, this study has provided a comprehensive and systematic examination of *D*. *arenosum*. The new species demonstrated adaptations to arid conditions, as evidenced by its life cycle and pH levels. The identification of *D*. *arenosum* contributes to the expanding knowledge regarding myxomycete diversity in arid regions, where these organisms were previously perceived as rare in desert environments. The rapid formation of swarm cells and sclerotia in *D*. *arenosum* likely constitutes a crucial adaptation for coping with environmental fluctuations. Overall, this study not only deepens our understanding of the life cycle and survival strategies of these organisms but also emphasizes the importance of ongoing exploration in arid regions to unveil the diversity and distinctive adaptations of various protist groups, such as myxomycetes.

##  Supplemental Information

10.7717/peerj.16725/supp-1 Supplemental Information 1 Sequences

## Additional Information and Declarations

### DNA Deposition

The following information was supplied regarding the deposition of DNA sequences: The new species sequences are available at GenBank: MW413352, MN720571. The *Didymium crustaceum* are available at GenBank: MW404616, MW404618, MW404630, MW404631. The *Didymium nigripes* are available via GenBank: MW404632.

### Data Availability

The following information was supplied regarding data availability: The raw sequences are available in the Supplemental File.

### New Species Registration

The following information was supplied regarding the registration of a newly described species: Mycobank: MB849855
